# SIAM (Suicide intervention assisted by messages): the development of a post-acute crisis text messaging outreach for suicide prevention

**DOI:** 10.1186/s12888-014-0294-8

**Published:** 2014-11-18

**Authors:** Sofian Berrouiguet, Zarrin Alavi, Guillaume Vaiva, Philippe Courtet, Enrique Baca-García, Pierre Vidailhet, Michel Gravey, Elise Guillodo, Sara Brandt, Michel Walter

**Affiliations:** Brest Medical University Hospital at Bohars, Adult Psychiatry, 29820 Bohars, France; INSERM Clinical Investigation Center 1412, Brest Medical University Hospital, Bohars, France; Clinical Investigation Center 9301, INSERM et CHU Lille, Lille, France INSERM U888; Montpellier University Hospital, INSERM U1061 Montpellier, France; Department of Psychiatry at Fundación Jimenez Diaz Hospital, Autónoma University, CIBERSAM, Madrid, 28040 Spain; Strasbourg Medical University Hospital, Adult Psychiatry, 67000 Strasbourg, France; Department of Psychology, Skidmore College, 815 North Broadway, Saratoga Springs, NY 12866 USA; Lanestel, 38 rue Jim Sévellec, 29200 Brest, France

**Keywords:** Prevention and control, Recurrence, Suicide, attempted, Telephone, Tertiary prevention, Text messaging

## Abstract

**Background:**

Suicidal behaviour and deliberate self-harm are common among adults. Research indicates that maintaining contact either via letter or postcard with at-risk adults following discharge from care services can reduce reattempt risk. Feasibility trials demonstrated that intervention through text message was also effective in preventing suicide repetition amongst suicide attempters. The aim of the current study is to investigate the effect of text message intervention versus traditional treatment on reducing the risk of suicide attempt repetition among adults after self-harm.

**Methods/design:**

The study will be a 2-year multicentric randomized controlled trial conducted by the Brest University Hospital, France. Participants will be adults discharged after self–harm, from emergency services or after a short hospitalization. Participants will be recruited over a 12-month period. The intervention is comprised of an SMS that will be sent at h48, D7, D15 and monthly. The text message enquires about the patients’ well-being and includes information regarding individual sources of help and evidence-based self help strategies. Participants will be assessed at the baseline, month 6 and 13. As primary endpoint, we will assess the number of patients who reattempt suicide in each group at 6 months. As secondary endpoints, we will assess the number of patients who reattempt suicide at 13 month, the number of suicide attempts in the intervention and control groups at 6 and 13 month, the number of death by suicide in the intervention and control groups at month 6 and 13. In both groups, suicidal ideations, will be assessed at the baseline, month 6 and 13. Medical costs and satisfaction will be assessed at month 13.

**Discussion:**

This paper describes the design and deployment of a trial SIAM; an easily reproducible intervention that aims to reduce suicide risk in adults after self-harm. It utilizes several characteristics of interventions that have shown a significant reduction in the number of suicide reattempts. We propose to assess its efficacy in reducing suicide reattempt in the suicide attempter (SA) population.

**Trial registration:**

The study was registered on Clinical Trials Registry (clinicaltrials.gov): NCT02106949, registrerd on 06 June 2014.

## Background

The construction of effective suicide-prevention and continuity of care strategies is a major challenge in the assessment and management of suicide attempters (SAs). Post-crisis management of suicidal patients varies from hospital discharge to forced hospitalization. Hospitalization rate after suicide attempt varies from 25 to 50% [1]. Fourteen to 25% of SAs treated at an emergency department (ED) reattempt suicide within the 1–2 years following discharge [2,3].

Outreach monitoring interventions via mail or phone facilitate access to care in case of recurrence in post-acute crisis patients discharged from the ED. Several studies on outreach interventions for suicidal patients have been published since 2001. The pioneer intervention was proposed by Motto et al. [4] and was based on postal contact. They proposed a follow-up in fifteen years after having started the inclusions in 1969. The objective of the postal contact was to show the patient that someone was concerned about his or her situation and maintain positive feelings towards him or her. After 5 years, a significant decrease in suicide related deaths was observed in the contacted group (versus no-contact group) of high-risk patients who refused on-going treatment. Carter et al. [5] programmed automated sent postcards. In the year following the suicide attempt, the authors reported a lower number of relapse in the contacted group, especially among women.

These positive results led to other studies exploring the effectiveness of telephone outreach (Vaiva et al. [6]). The telephone contact at one month proved, in per protocol analysis, to reduce the number of suicidal reattempts by about half over the course of one year (12% versus 21.6% in the control group). The post-hoc analysis showed no effect of the contact in first-attempters. The proposal of telephone contact was very well accepted and positively perceived by the population of this study.

In the EDGE project, a hotline was available to participants 24 hours a day, 7 days a week [7]. In the intervention group, suicide attempters repeaters rate did not differ significantly from that in the control group (15.1% vs. 17.3% at one year, 21.2% vs. 22.8% at two years). However, there was a lower number of reattempts in intervention group among suicide attempters repeaters (incidence risk ratio 0.55 at one year, and 0.49 at two years).

Vaiva and Walter proposed to construct a case management algorithm [8]. This «ALGOS» monitoring algorithm is based on the two interventions that showed a significant reduction in the number of SA repeaters: systematic telephone contact (ineffective in first-attempters) and «crisis card» (information card providing a 24/7 reachable phone number, effective only in first-attempters). Participants that were not contacted during phone call periods and participants that refused proposed healthcare can benefit from the «short letters» of Motto or the «postcards» of Carter.

A Chinese study published in 2010 proposed to use a different type of intervention that involved sending text messages to suicidal patients after discharge from the ED [9]. They sent 15 patients four text messages for four weeks following a suicide attempt. The text of the message varied for each transmission but the four text messages were the same for all patients. Although this type of intervention proved to be effective, management of large patient populations was not possible with the proposed device.

Taking into consideration the strengths and pitfalls of each of these strategies, we developed a software program that allows the transmittance of text messages to SAs. The SIAM (suicide intervention assisted by messages) software that we developed aims to simplify the inclusion of the patients and to have an optimized text sending. We assessed the technical feasibility and acceptability of this device in a pilot study [10]. Text messages provide several characteristics of existing monitoring strategies for suicide attempt repeaters. Using our software, we personalized the messages as recommended by Motto [4] and reached patients earlier than with a letter. Our software program allows us to readily personalize each message according to the required level of care. We clearly identify care services as the senders of the messages, as would have been done in a letter. Call-back numbers proposed in our messages are available 24 h per day and 7 days per week, in line with the contact strategy proposed by Evans et al. [7]. In comparison to the contact strategies by letter, access to our service was made easier as the patient simply had to press the call button on his/her telephone to contact us upon receiving the text message. Furthermore, sending a message with our device is 10 times cheaper than sending a postcard or a letter. The utilization of this monitoring system does not require the action of a clinician. All messages are written and scheduled before discharge and automatically sent over the monitoring period. Despite the variation in suicidal behaviour across the population [11], the worldwide dispersion of mobile phones could offer new perspectives in suicide reattempt prevention. As text messages utilize several characteristics of interventions that have significantly reduced the number of suicide reattempts, we propose to assess its efficacy on reducing suicide reattempt in SA population.

## Methods/design

### Aims and hypotheses

This study aims to determine whether the receipt of a text message sent regularly over a six-month period can reduce suicidal and self-harming behaviour among SAs.

We hypothesize that the routine reception of a text message during a six-month period will:

1. Decrease the proportion of reattempters at 6 and 13 months;
2. Decrease the number of total reattempts at 6 and 13 months;
3. Decrease suicide deaths;
4. Decrease suicidal ideations;
5. Decrease intervention costs;
6. Improve satisfaction through care services.

### Study design

The study is a 2-year multicentric randomized controlled trial conducted by the Brest University Hospital, France. Participants are adults discharged after self–harm, recruited in Brest, Lille, Rennes, Nantes, Tours, Vannes, and Saint-Nazaire. Participants will be recruited over a 12-month period. The study is a randomized controlled trial registered on Clinical Trials Registry (ClinicalTrials.gov; number: NCT02106949). It was authorized by ANSM (French Health Ministry) and approved by the Northwest IV Ethical Committee for the Protection of Patients.

### Setting

Participating centers are Brest, Rennes, Nantes, Lille, Angers, Tours, Vannes, (France) - public funded specialist mental health services for adults in the Western and Northwestern regions of France.

### Participants

Inclusion criteria are: male or female, aged 18 or older, surviving a suicide attempt, discharged from ED or Psychiatric Units (PU), hospitalized for less than 7 days, giving consent, and able to be contacted by phone.

The exclusion criteria are: refusing to participate, underage, incarcerated, under guardianship, without a mobile phone, enrolled in other trials, and in emergency situations where their state of health did not allow obtaining written consent.

In the case of discharge, a follow-up visit or planned hospitalization will be scheduled.

### Procedure

Participation in the study will be proposed to all suicidal adults referred to our psychiatric ED meeting the above criteria. In each center, adults who attempt suicide are admitted to the general ED and are evaluated by our ED psychiatrist who decides patients’ discharge or hospitalization. Patients will be enrolled after this evaluation. After enrolment, patients will be assigned to the intervention or control group, using an electronic randomization program. At month 6 and month 13, patients in both intervention and control groups will be contacted by phone for the evaluation. A trained psychiatrist will administer the evaluation.

### Intervention

Text messages are customized with each patient’s name and sent with an identical outreach schedule for each patient. We will send nine different text messages to each patient 48 hours after discharge, at day 8, day 15 and month 1, 2, 3, 4, 5, 6. All the text messages will be sent at 1 pm. The four messages will refer to: the validation of the suffering, recall of the discharge agreement, and the monitoring system, i.e. our outreach continuing care intervention.

They also will include information in regard to the monitoring doctor’s name (psychiatrist or general physician), and the date of the scheduled appointment if applicable. The contact phone number will be indicated in each text message transmission and the message will appear as sent from the psychiatric emergencies hotline number reachable 24/7. One example of the messages received by the patients will be: *“Mr X, we hope that your situation is getting better and that you could go to the Dr Y consultation (April 7th 2011 at 10 h00). You can call us for anything you may need at 0298000000”.* A final reminder text message will be sent 48 hours before the call-back telephone evaluation of month 6 and 13. Patients will be followed up by the psychiatrist of their choice or by their general physician. Text messaging monitoring will be proposed as an additional support to their standard of care.

#### Control intervention

Those in the control group will receive the follow up as usual including psychiatrist or physician consultation.

### Outcomes measures

As primary endpoints, we will assess the number of patients who will reattempt suicide in each group at month 6.

As secondary endpoints, we will assess the number of patients who will reattempt suicide at month 13, the number of suicide attempts in the intervention and control groups at month 6 and 13, and the number of deaths by suicide in the intervention and control groups at 6 and 13 month. In both group, suicidal ideations will be assessed at the baseline, 6 and 13-months. Medical costs and satisfaction will be assessed at month 13.

Baseline evaluation will be done in ED or PU before discharge. Socio-demographic details, including age, gender, employment, educational status, history of suicide attempt, country of birth, medical history and details of any treatment being received, will be recorded on a specifically designed, standardized questionnaire.

The Columbia Scale measures suicidal ideation and intent. It is a 25 item screening scale with items including binary variables (yes or no) and variables scales for each question. [12]. It is also available in French.

Axis-I psychiatric diagnoses will be assessed using the International Neuropsychiatric Interview (M.I.N.I.). It is a short, structured diagnostic interview developed for DSM-IV and ICD-10 psychiatric disorders [13]. With an administration time of approximately 15 minutes, it is the structured psychiatric interview of choice for psychiatric evaluation and outcome tracking in clinical psychopharmacology trials and epidemiological studies. It has been validated in French [14].

The medico-economic questionnaire (MEDEC) is a use of care services inventory including binary variables. It is carried out by the method validated by Beecham & Knapp at 13 month [15].

The satisfaction questionnaire was designed by our team [10] to assess whether such outreach system was accepted by the patients. The subjective system effectiveness will be explored to assess patients’ acceptability.

### Sample size calculation: effect size and statistical power

Expected reattempt rate is about 18% [6,16]. We hope to reduce this rate to 9% in the intervention group. Such a reduction in reattempts with a statistical power of 0.8, requires a sample size of 247 patients in each group. Taking into account the expected proportion of SAs that will not respond to the follow-up message, 530 patients in total will be recruited.

### Randomisation/intervention

Random allocation to the intervention group and the control group will be carried out by an independent statistician and will be stratified by center study using blocked randomization and computer generated random numbers. This will be conducted using an online randomization system. This will be concealed from the research team (psychiatrist, nurse). The statistician will notify the study coordinator regarding the group allocation. Text messages will be sent by SIAM program, designed for the pilot study. Once the patient data is entered, the program will generate four independent text messages containing patient’s name, doctor's name and monitoring schedule from the date of inclusion. At any time the patient data, date of sending the text message and message content could be modified. At any time, sending messages (transmissions) could be interrupted. The number displayed on the recipient’s mobile phone was the psychiatric ED.

### Statistical methods

Continuous variables will be summarized using mean, standard deviation, median and inter-quartile range (IQR). Categorical variables will be summarized using frequency, percentage and 95% CI. Means will be compared between the intervention and control groups using the Student test (t-test) or Mann–Whitney test. Comparisons of proportions between groups will be performed using the Chi square test or Fisher’s exact test as appropriate.

Generalized linear model will be used to compare the intervention and control groups for each outcome measure over time (baseline, 6 months, 13 months). The corresponding baseline values of each outcome measure will be used as a covariate. In addition, the effects of other possible predictors (such as gender and age) on outcome will be explored.

Analysis of predictive factors of suicide attempt repetition will be performed using logistic regressions. An approach by a decisional tree (CHAID) will also be considered. Analysis of time delay of SA repetition will be performed using conventional methods of survival analysis: Kaplan-Meyer method, log-rank test and Cox model for multifactorial models. The research for specific profiles will be conducted using classification methods to identify clusters with atypical profiles.

An interim analysis will be conducted on the first 250 participants using the O’Brian and Fleming method. If the difference between intervention and control groups is not significant (p <0.5%), statistical significance will have to reach a level of p < 4.8% by the end of the study to demonstrate a significant difference. A Data and Safety Monitoring Board (DSMB) will conduct this assessment.

### Safety and supervision

Patients are informed that they can contact the center where they have been treated at any time. Phone contact will be established by a trained psychiatrist and research assistant at month 6 and 13 to assess the outcome measures. If there is no cause for concern after the 6 month phone call evaluation, patients from each group will be informed that they will be contacted at month 13 for a new evaluation. If there are concerns about the participant, contact will be made by phone or face-to-face as to discuss the concerns. If the research fellow is concerned about the participant, an immediate referral will be made to an appropriate service. Any patient considered to be at high risk will be referred to an appropriate service.

## Discussion

This paper describes the protocol for a Randomized Clinical Trial that aims to reduce reattempt among SAs. Postal and phone interventions have shown efficacy in prevention of suicide reattempts [4,5,7].

This low-cost and transferable SMS intervention has the potential to reach a large number of people who are traditionally hard to reach and treat. Non-compliant patients, especially young people, could be an interesting target of such a monitoring device. The SMS allows the health care team to keep in touch with people that are not reachable by existing contact strategies. This intervention is not a substitute for treatment. It may be used as a useful additional or alternative to standard care when people refuse other forms of treatment. Even though we used a web system to deploy our text monitoring device, our main goal is to encourage human to human contact between SAs and health care providers. The absence of social relationship cannot be completely replaced by a text message care providing service. However, it is essential for ED and PU staff to maintain contact with patients after a suicide attempt. The beneficial effects of this therapeutic monitoring intervention can be utilized as a long-term follow-up for SAs by allowing them to feel continuously cared for by the PU and ED staff. We believe that this monitoring tool can be used shortly after discharge from medical care centers in prevention of high-risk post-acute suicide. The use of this type of suicide intervention tool also lends itself to the development of more complex programs such as a mobile app or intervention website. These additional resources may also offer effective opportunities to maintain contact with SAs.
